# Marker-controlled watershed algorithm and fuzzy C-means clustering machine learning: automated segmentation of glioblastoma from MRI images in a case series

**DOI:** 10.1097/MS9.0000000000001756

**Published:** 2024-01-26

**Authors:** Sadegh Ghaderi, Sana Mohammadi, Kayvan Ghaderi, Fereshteh Kiasat, Mahdi Mohammadi

**Affiliations:** a Department of Neuroscience and Addiction Studies, School of Advanced Technologies in Medicine, Tehran University of Medical Sciences, Tehran; b Department of Medical Sciences, School of Medicine, Iran University of Medical Sciences, Tehran; c Department of Information Technology and Computer Engineering, Faculty of Engineering, University of Kurdistan, Sanandaj; d Department of Medical Physics and Biomedical Engineering, School of Medicine, Tehran University of Medical Sciences, Tehran, Iran

**Keywords:** GBM, marker-controlled watershed algorithm, MRI, tumour segmentation

## Abstract

**Introduction and importance:**

Automated segmentation of glioblastoma multiforme (GBM) from MRI images is crucial for accurate diagnosis and treatment planning. This paper presents a new and innovative approach for automating the segmentation of GBM from MRI images using the marker-controlled watershed segmentation (MCWS) algorithm.

**Case presentation and methods:**

The technique involves several image processing techniques, including adaptive thresholding, morphological filtering, gradient magnitude calculation, and regional maxima identification. The MCWS algorithm efficiently segments images based on local intensity structures using the watershed transform, and fuzzy c-means (FCM) clustering improves segmentation accuracy. The presented approach achieved improved segmentation accuracy in detecting and segmenting GBM tumours from axial T2-weighted (T2-w) MRI images, as demonstrated by the mean characteristics performance metrics for GBM segmentation (sensitivity: 0.9905, specificity: 0.9483, accuracy: 0.9508, precision: 0.5481, F_measure: 0.7052, and jaccard: 0.9340).

**Clinical discussion:**

The results of this study underline the importance of reliable and accurate image segmentation for effective diagnosis and treatment planning of GBM tumours.

**Conclusion:**

The MCWS technique provides an effective and efficient approach for the segmentation of challenging medical images.

## Introduction

Highlights

- Development of a novel fully automated method for glioblastoma multiforme extraction using fuzzy c-means and marker-controlled watershed segmentation.
- Marker-controlled watershed segmentation combined with fuzzy c-means exhibited high sensitivity and specificity.
- The method is a promising approach for accurate and reliable segmentation.
- Faster response time compared to the traditional watershed methods.

Gliomas are a type of brain tumour that arises from glial cells^1^. There are different types of gliomas, including astrocytomas, oligodendrogliomas, and ependymomas, which can be categorized based on their specific cell type and histological characteristics^1–4^. Gliomas are further graded based on their aggressiveness, with grades I and II being considered low-grade and grades III and IV being high-grade^1,3,5,6^.

Glioblastoma multiforme (GBM) is the most aggressive and malignant form of glioma, having a grade IV rating. GBM is one of the most lethal forms of primary brain cancer^7^. Surgery is often the first-line treatment for GBM, with the aim of removing as much of the tumour as possible without damaging critical brain tissue. The extent of surgical removal will depend on the size and location of the tumour, as well as the patient’s overall health and neurological function^8^. However, complete removal of the tumour is often not possible, as GBM tends to infiltrate the surrounding brain tissue^8,9^. Accurate detection and segmentation of GBM tumours from MRI images are crucial for effective diagnosis, treatment planning, and the prediction of patient outcomes^10^.

MRI is the most commonly used imaging modality for GBM detection, particularly axial T2-weighted (T2-w) images without contrast media, as contrast media can present adverse effects and potential risks for patients. However, the visual interpretation of MRI images can be challenging due to the complexity and heterogeneity of GBM tumours, particularly in subregions of the brain with high variability in tissue contrast, such as the periventricular areas^11–14^.

Over the past two decades, image processing algorithms have become increasingly important for improving the accuracy and reliability of GBM detection and segmentation from MRI images^15,16^. For the first time, our study uses particularly the marker-controlled watershed segmentation (MCWS) algorithm in GBM segmentation. This algorithm uses markers to guide the segmentation process and assigns labels to different regions based on the local intensity gradient^17–19^. This allows for better differentiation between different regions of the tumour and for more accurate localization of the tumour borders beyond those defined by the manual segmentation of physicians^17,20^. We propose a novel approach to automatically detecting GBM using MRI images by utilizing the MCWS algorithm. The MCWS algorithm’s efficiency lies in its ability to segment images efficiently using the watershed transform, and fuzzy c-means clustering improves segmentation accuracy, relying on several image processing techniques, including adaptive thresholding, morphological filtering, gradient magnitude calculation, and regional maxima identification^21,22^. The algorithm is compared with state-of-the-art methods, providing a new framework for more accurate and automated GBM detection. The study highlights the significance of reliable image segmentation for precise diagnosis and treatment planning of GBM tumours. The proposed framework proves to be an effective and efficient approach for the segmentation of challenging medical images.

Eventually, this paper proposes a new and effective methodology for GBM detection and segmentation that combines axial T2-w MRI images, image pre-processing techniques, and the MCWS algorithm. The proposed methodology aims to provide fast and more accurate segmentation of GBM tumours, including deep infiltration and necrotic regions, and to minimize the human bias of manual segmentation methods.

## Methods

### Patient’s history

**Figure 1:**
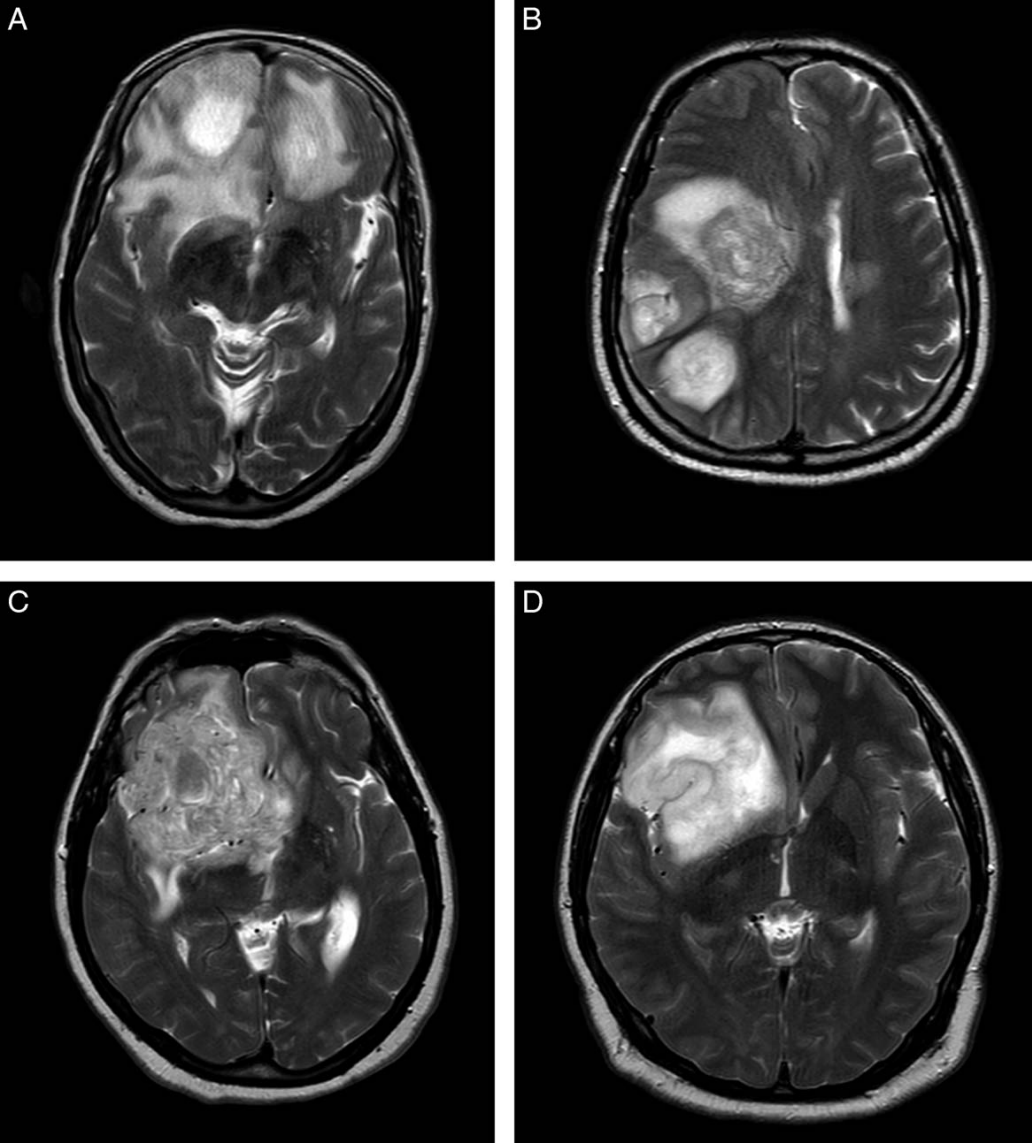
Axial T2-weighted MRI images of glioblastoma multiforme (GBM) in four patients: (A) Multi-focal GBM lesions in the right and left frontal lobes of an 81-year-old female (patient 1). (B) Multi-focal intracranial GBM lesions in a 62-year-old male (patient 2). (C) A right lateral ventricular and right frontal lobe GBM with mass effect in a 63-year-old female (patient 3). (D) A right frontotemporal GBM in a 32-year-old male (patient 4).

This case series has been reported following the 2020 guidelines for Preferred Reporting of Case Series in Surgery (PROCESS)^23^, Supplemental Digital Content 1, http://links.lww.com/MS9/A364 and Surgical Case Report (SCARE)^24^. The patient descriptions in Table 1 provide crucial information about the demographics, medical history, and tumour characteristics of those diagnosed with GBM. The proposed methodology was evaluated using four patient cases, including an 81-year-old female with a history of hypertension and head trauma. She was diagnosed with a high-grade tumoral process, namely multi-focal GBM, which was located in both the right and left frontal lobes (Fig. 1A), a 62-year-old male with a history of diabetes mellitus and hypertension, who was diagnosed with multi-focal GBM in the form of intracranial lesions (Fig. 1B), a 63-year-old female who presented with headaches, seizures, and cognitive deficits. She was diagnosed with high-grade tumoral prosses, namely GBM with mass effect, in the right lateral ventricle and the right frontal lobe (Fig. 1C), and a 32-year-old male with a family history of brain tumours, who was diagnosed with high-grade tumoral prosses, namely GBM, located in the right frontotemporal region (Fig. 1D).

**Table 1:** Demographics and medical history of GBM patients.

Patient number	Age	Sex	Previous medical history	Tumour grade	Tumour location
1	81	F	Hypertension and head trauma	High-grade tumoral prosses (multi-focal GBM)	Right and left frontal lobes
2	62	M	Diabetes mellitus and hypertension	Multi-focal GBM	Intracranial lesions
3	63	F	Headaches, seizures, and cognitive deficits	High-grade tumoral prosses (GBM) and mass effect	Right lateral ventricle and right frontal lobe
4	32	M	Family history and head injuries	High-grade tumoral prosses (GBM)	Right frontotemporal region

F, female; GBM, glioblastoma multiforme; M, male.

The axial T2-w images depicted in Figure 1 were selected at random from patients diagnosed with GBM, based on their depiction of the tumour area in the brain. Axial T2-w MRI imaging is a powerful and routine section for visualizing the internal brain structure and tissues, including the detection of tumours. These images were chosen to showcase the different locations and appearances of GBM tumours in the brain, as described in Table 1. Each image shows the unique characteristics of GBM, such as multifocality and mass effect. The selection of these specific images was made without any predetermined criteria or biases, ensuring that they represent a truly random selection of GBM cases.

### Marker-controlled watershed segmentation algorithm

GBM is the most common and aggressive form of primary brain cancer, with high rates of morbidity and mortality. While GBM is the most lethal and aggressive type of primary brain cancer, it can also be metastatic, which means that it has the potential to spread to other organs or body tissue. Accurate detection and segmentation of GBM tumours from MRI images are crucial for effective diagnosis and treatment planning, as well as the prediction of patient outcomes. Furthermore, GBM tumours often exhibit complex and heterogenous morphology, including infiltrative and necrotic regions, which poses a significant challenge for visual examination by radiologists^25–27^. In this context, image processing techniques play an important role in improving the accuracy and reliability of GBM tumour detection and segmentation from MRI images, particularly the MCWS Algorithm.

In our study, all images were acquired using a 1.5T MR scanner (Philips Ingenia, Philips Healthcare, Best, The Netherlands). This unit was equipped with an 8‑channel coil for brain examination. The study used axial T2-w MRI brain images acquired from four patients diagnosed with GBM. In this paper, based on a previous study done by Ghaderi *et al.* ^17^ we have used the MCWS technique for GBM segmentation in MRI images using MATLAB (r2022b) software (Natick).

MCWS algorithm is a powerful image segmentation algorithm used in computer vision and image processing. It can be broken down into the following five steps^18^:

1. Compute a segmentation function. This function should identify the objects of interest and create an image where the dark regions are the objects being segmented.
2. Compute foreground markers. These markers are connected blobs of pixels within each of the objects. They should be placed at the centre of each object to guide the segmentation process.
3. Compute background markers. These markers are pixels that are not part of any object. They should be placed in the areas around the objects to guide the segmentation process.
4. Modify the segmentation function so that it only has minima at the foreground and background marker locations. This is accomplished by placing a constraint on the function to ensure that the markers are respected during the segmentation process.
5. Compute the watershed transform of the modified segmentation function. This process will help segment the objects accurately and efficiently by dividing the image into regions based on the catchment basins created by the markers.

By following these steps, the MCWS algorithm achieves better segmentation results by incorporating the markers into the watershed segmentation algorithm. It is widely used in medical image analysis, object tracking, and video analysis^18,28,29^. The MCWS algorithm is a powerful technique for segmenting images, especially those with complex structures, such as medical images like MRI^17^. By using markers to guide the segmentation process and assigning labels to different regions based on the local intensity gradient, the algorithm can accurately identify and differentiate the different regions of an object in an image. We recommend following the steps of the MCWS algorithm, which are based on our previous work^19^.

A thematic map is a visual representation that displays a particular theme or feature related to a specific geographic area using colours, symbols, or other visual elements. It is commonly used to represent data related to topics such as population density, topography, soil types, climate, and more. In the context of imaging processing and analysis, a thematic map can represent segmented regions of an image by assigning unique colours or labels to different regions based on their pixel characteristics. It is commonly used in medical imaging to identify different types of tissues or structures in an image and assist in the diagnosis of medical conditions^30^.

In summary, the following MATLAB code reads in DICOM medical imaging files and applies various image processing techniques to segment and extract objects of interest from the image. The code first applies Otsu’s method for thresholding to binarize the input image. Then, it applies FCM clustering to generate two thresholded images and displays the results. The code then applies adaptive thresholding and filters objects based on area. The area, eccentricity, diameter, Euler number, major and minor axis length, orientation, and perimeter properties of the remaining objects are calculated and sorted by area. The code calculates the gradient magnitude image and applies the watershed transform to segment the image based on gradient magnitude. Morphological operations are performed, regional maxima are identified, and watershed segmentation is applied to label regions of the image. The output of the algorithm is the original image overlaid with markers and object boundaries to display regions of interest in the image. Automatic segmentation methods can provide faster and more consistent results but require careful parameter tuning and validation. Markers (also known as seeds or initial points) are placed in the segmented tumour region to guide the watershed transformation. These markers indicate the areas of the image that correspond to the tumour and are used as a starting point for the algorithm.

Automatic marker placement can be done based on regional maxima or using other methods such as clustering or machine learning. A distance transform is applied to the tumour region to generate a map of the intensity gradient, which is used to distinguish between different regions of the tumour. The distance transform assigns a distance value to each pixel in the tumour region based on its distance to the nearest background pixel. This allows the watershed algorithm to segment the tumour into distinct regions according to the intensity gradient. The MCWS transform is performed using the distance transform as the input image. This produces a segmentation of the tumour region into different subregions based on the local intensity structure. The watershed algorithm starts at the markers and floods the image with labels until it reaches boundaries defined by the intensity gradient. The resulting segmentation divides the tumour into regions defined by the gradient and the markers. Finally, the coloured watershed label matrix is superimposed transparently on the original image to provide an informative visual representation of the segmented objects in the image.

## Results

In image segmentation, the goal is to segment an image into meaningful regions or objects^31^. One common method to achieve this is by using thresholding techniques. In this algorithm, the first step in the image segmentation algorithm is to import a DICOM medical image file of Axial T2-w MRI images containing GBM tumours. The image must have sufficient resolution and contrast for accurate tumour segmentation. The second step in the algorithm is to convert the image to grayscale to simplify the processing and analysis steps. Otsu’s method is used in the third step to threshold the image, which automatically determines the optimal threshold value to separate the foreground from the background in the image. This technique is widely used in image segmentation because it can effectively partition an image into two classes with minimal human intervention.

**Figure 2:**
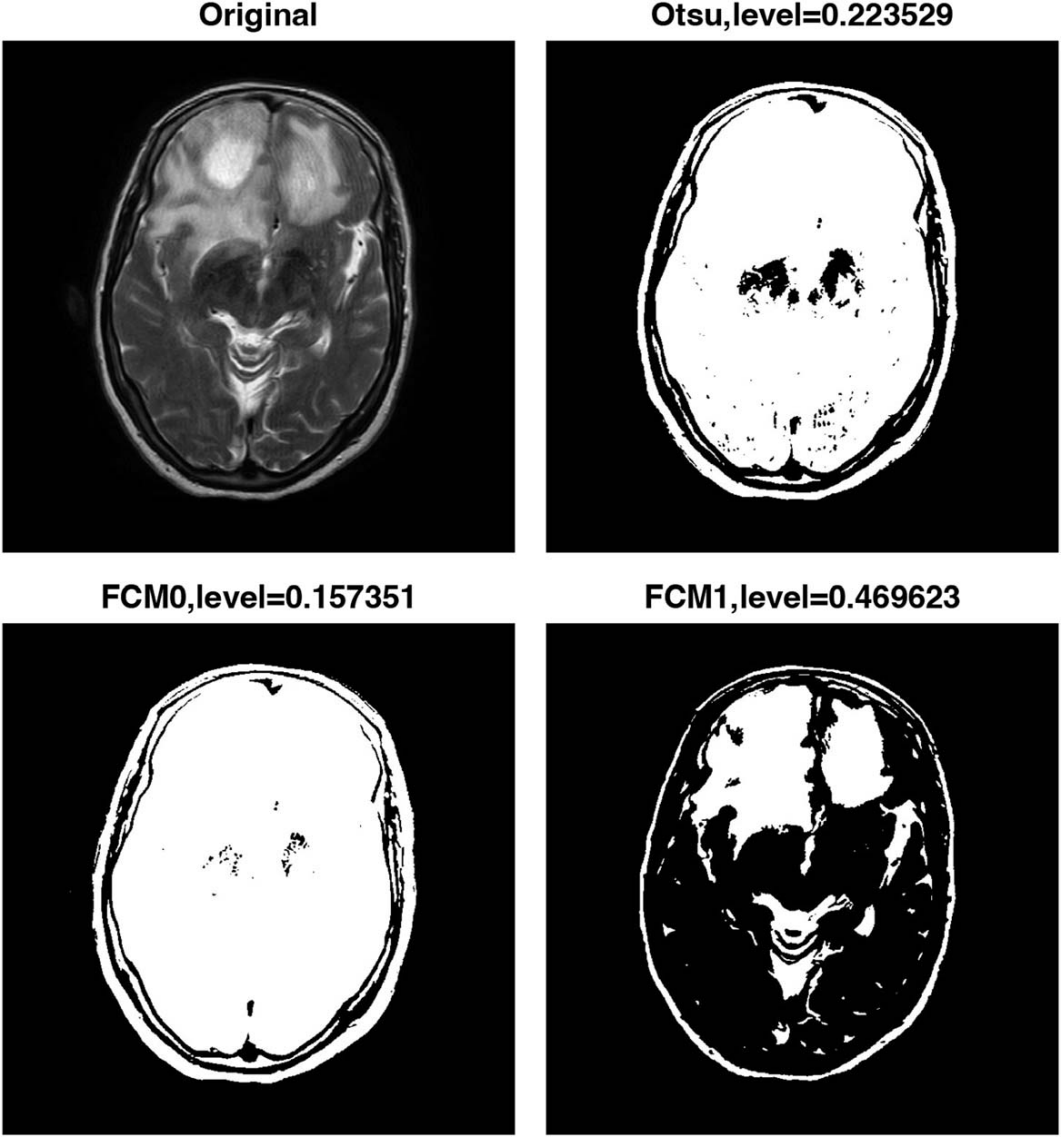
Pre-processing steps for segmentation of multi-focal glioblastoma multiforme tumours in axial T2-weighted MRI images of patient 1. FCM, fuzzy c-means.

**Figure 3:**
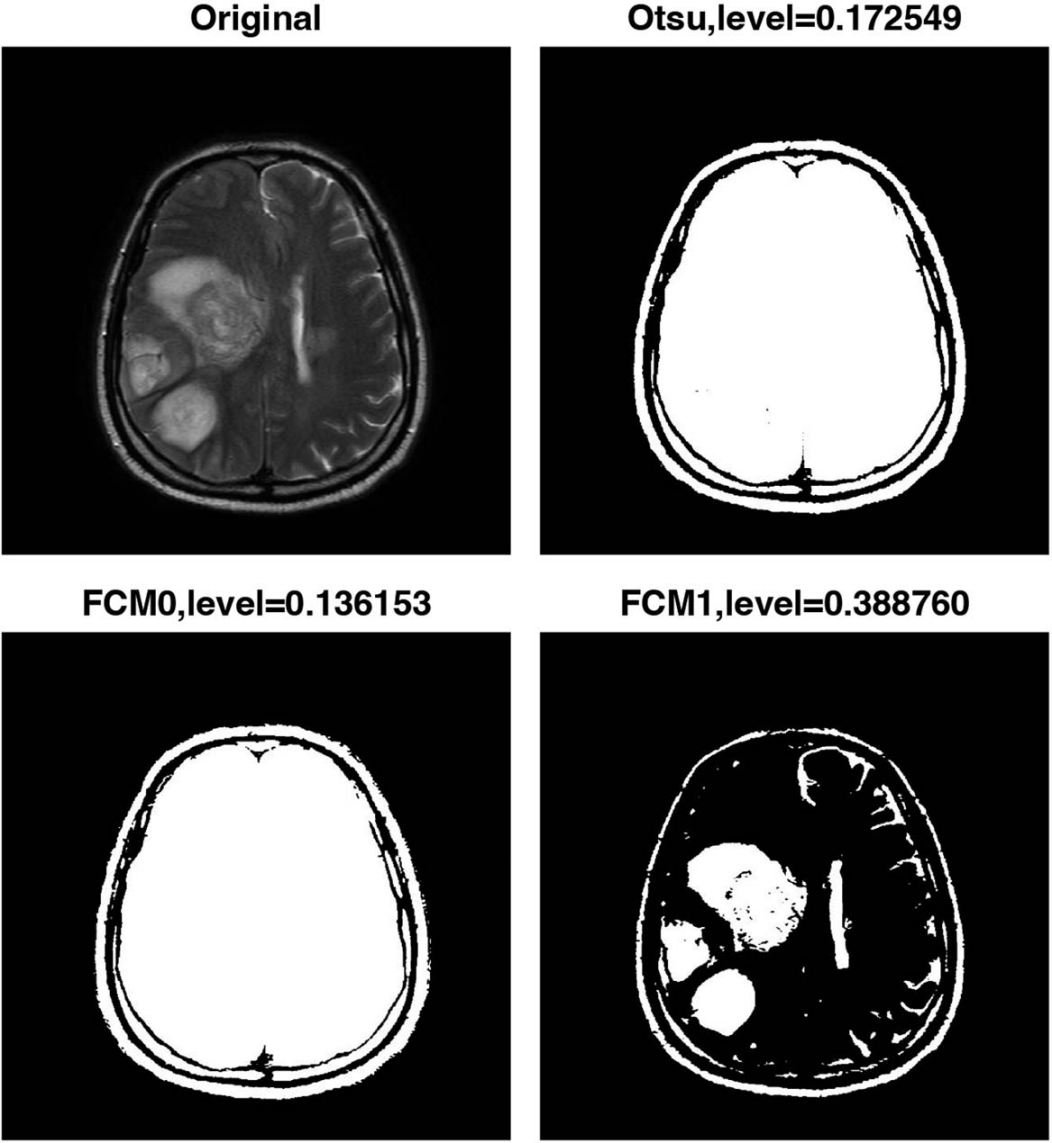
Pre-processing steps for segmentation of multi-focal glioblastoma multiforme tumours in axial T2-weighted MRI images of patient 2. FCM, fuzzy c-means.

**Figure 4:**
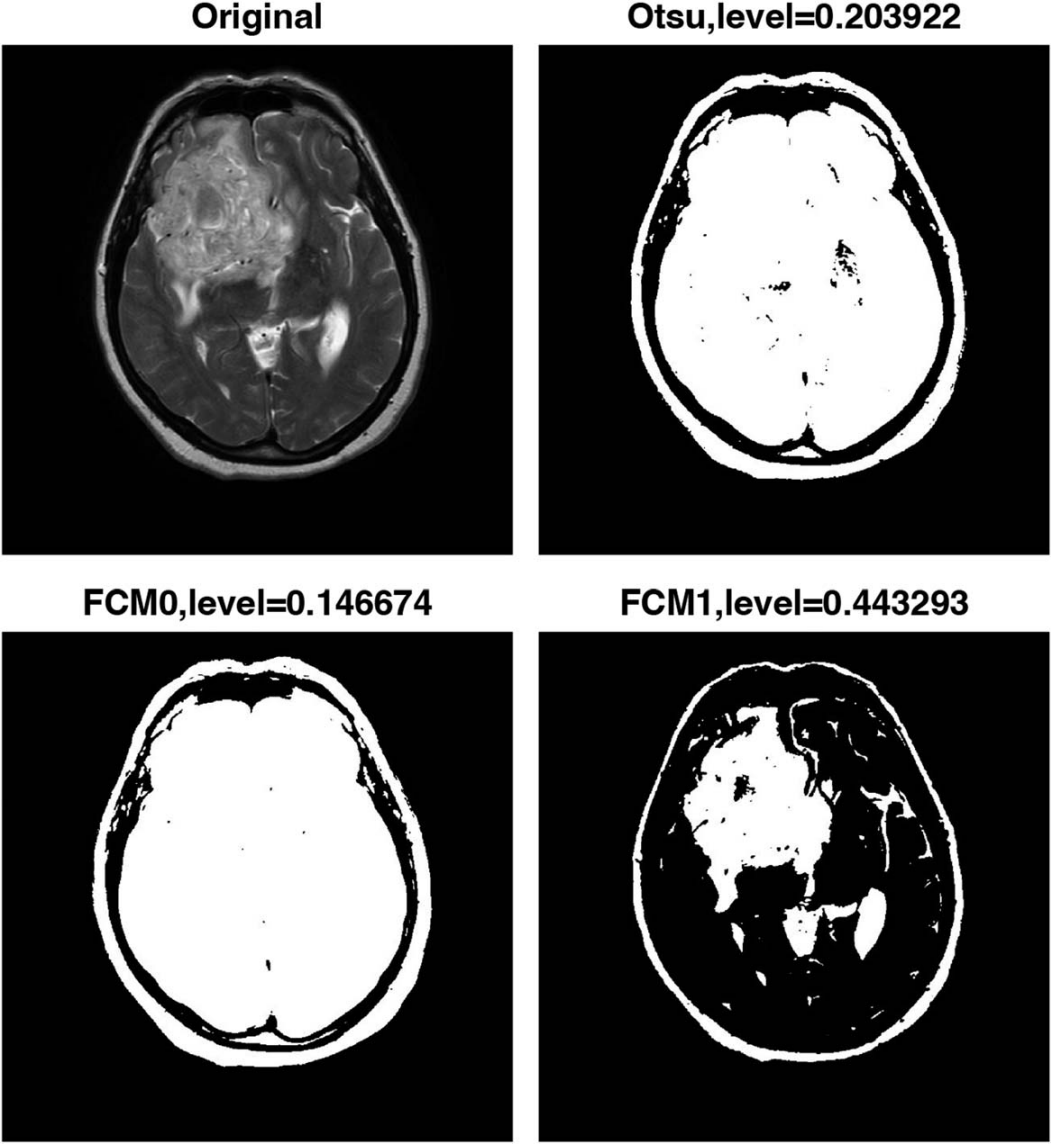
Pre-processing steps for segmentation of multi-focal glioblastoma multiforme tumours in axial T2-weighted MRI images of patient 3. FCM, fuzzy c-means.

**Figure 5:**
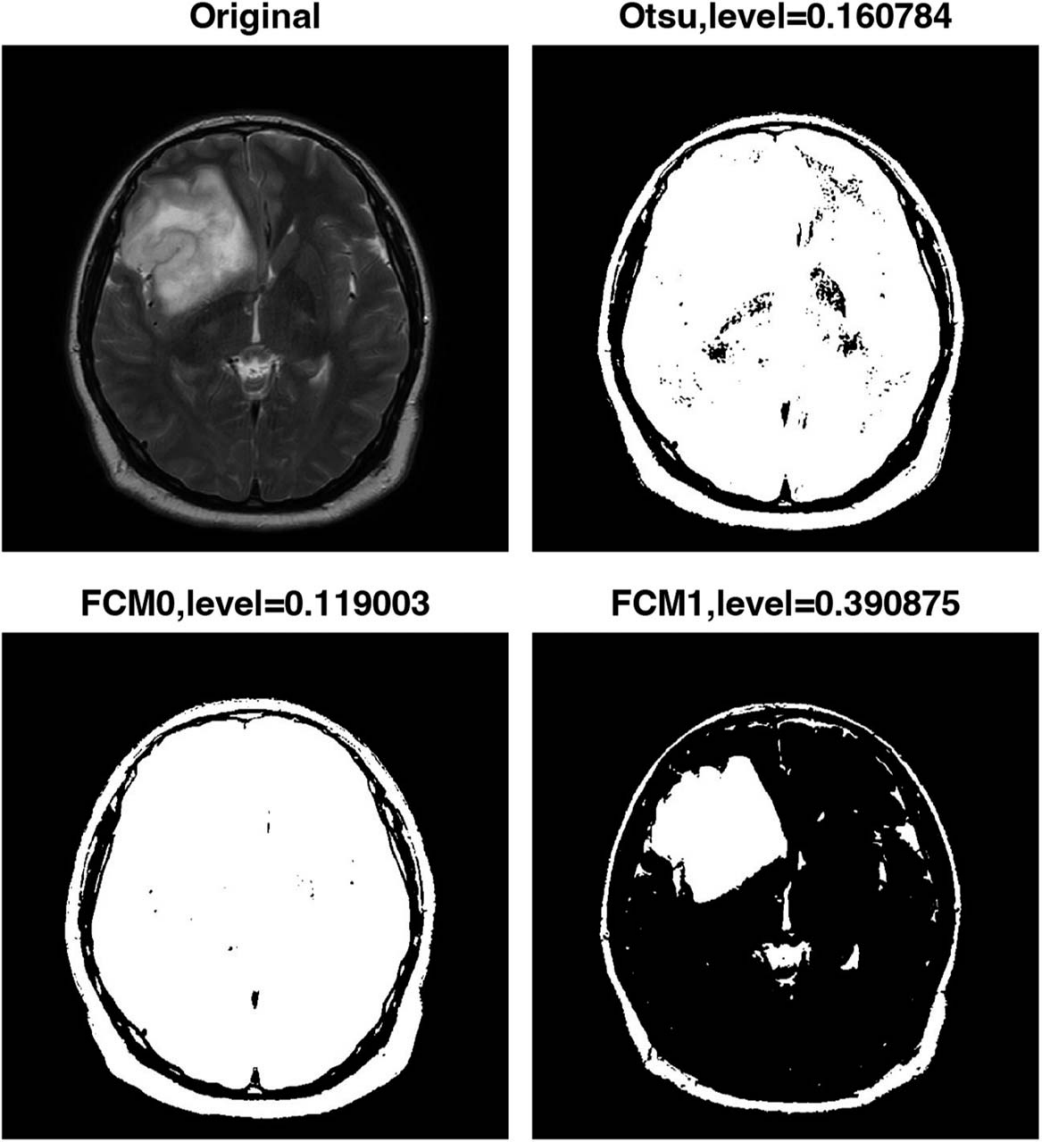
Pre-processing steps for segmentation of multi-focal glioblastoma multiforme tumours in axial T2-weighted MRI images of patient 4. FCM, fuzzy c-means.

Finally, the algorithm uses FCM clustering to generate two thresholded images based on similarity to further improve the accuracy of the segmentation. FCM clustering allows pixels to have membership in multiple clusters, resulting in more robust segmentation results. Figures 2–5 show the results of the image segmentation algorithm on an axial T2-w MRI image with a GBM tumour using Otsu’s method and FCM clustering for patients 1 through 4. The top left image is the original input image; the top right image is the thresholded image generated using Otsu’s; and the bottom left and right images are the thresholded images generated using FCM clustering. The segmentation accuracy is improved with the use of FCM clustering.

Table 2 displays the thresholding levels obtained using Otsu’s method and FCM clustering for each patient’s GBM tumour image, as well as the corresponding threshold levels for the two clusters generated by FCM clustering. The table also includes the dice similarity coefficient (DSC) values for each segmentation approach.

**Table 2:** Thresholding levels.

#	FCM threshold level 0	FCM threshold level 1	Otsu’s level	DSC (Otsu’s vs. FCM1)
Figure 2, Patient 1	0.157351	0.469623	0.223529	0.7028
Figure 3, Patient 2	0.136153	0.388760	0.172549	0.5595
Figure 4, Patient 3	0.146674	0.443293	0.203922	0.6892
Figure 5, Patient 4	0.119003	0.390875	0.160784	0.4782

DSC, dice similarity coefficient; FCM, fuzzy c-means.

For patient 1, the thresholding levels obtained using FCM clustering were 0.157351 and 0.469623, while the thresholding level obtained using Otsu’s method was 0.223529. For patient 2, the FCM clustering thresholding levels were 0.136153 and 0.388760, while the thresholding level obtained using Otsu’s method was 0.172549. For patient 3, the FCM clustering thresholding levels were 0.146674 and 0.443293, while the thresholding level obtained using Otsu’s method was 0.203922. Finally, for patient 4, the FCM clustering thresholding levels were 0.119003 and 0.390875, while the thresholding level obtained using Otsu’s method was 0.160784.

DSC stands for the dice similarity coefficient, which is a statistical measure that is commonly used to evaluate the performance of image segmentation algorithms. The DSC compares the similarity between two sets of data, such as a segmentation algorithm output and a reference standard, and is calculated by taking the ratio of the overlap between the two sets to the total number of pixels in both sets. The DSC is defined as:


DSC=2*(A∩B)/(|A|+|B|)

where A and B are the two sets of data being compared, and |A| and |B| represent the total number of pixels in each set. A ∩ B represents the number of pixels that are common to both sets or the overlap.

The DSC ranges from 0 to 1, where a value of 1 indicates a perfect overlap between the two sets, and a value of 0 indicates no overlap. A DSC score of 0.7 or higher is generally considered to be a good score in medical imaging applications. The results of using two different segmentation methods, FCM clustering, and Otsu’s method, were compared using DSC scores. The table shows the DSC scores for each method on four different patients. The FCM clustering consistently produced higher DSC values compared to Otsu’s method, which indicates better segmentation accuracy. This means that the FCM clustering method is likely a better choice for medical imaging applications.

**Figure 6:**
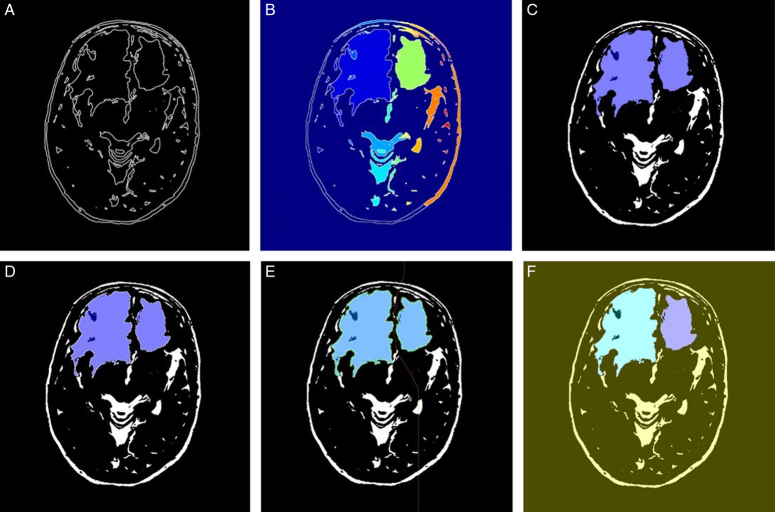
The marker-controlled watershed segmentation algorithm for glioblastoma multiforme segmentation (patient 1).

**Figure 7:**
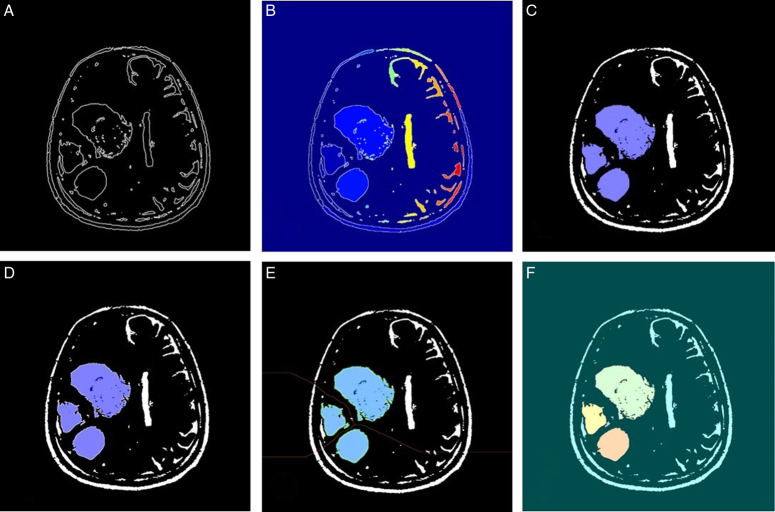
The marker-controlled watershed segmentation algorithm for glioblastoma multiforme segmentation (patient 2).

**Figure 8:**
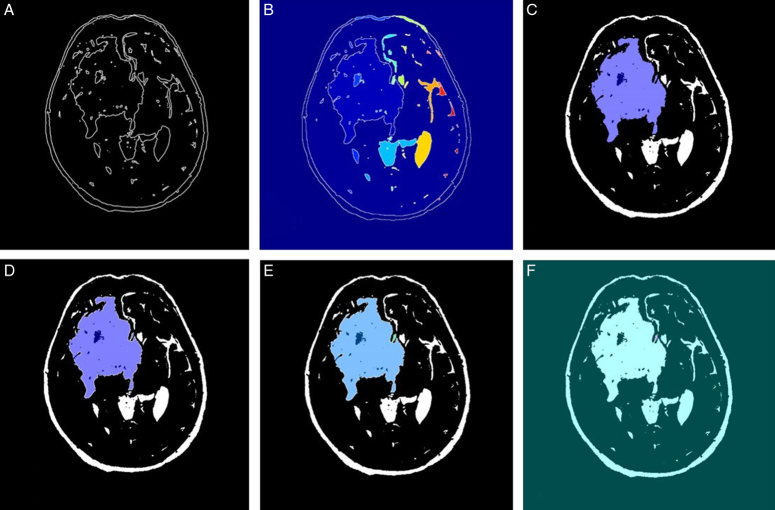
The marker-controlled watershed segmentation algorithm for glioblastoma multiforme segmentation (patient 3).

**Figure 9:**
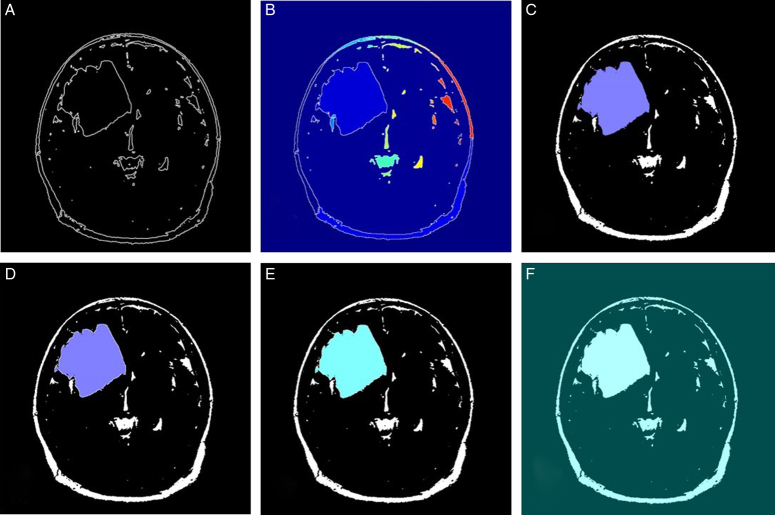
The marker-controlled watershed segmentation algorithm for glioblastoma multiforme segmentation (patient 4).

The given algorithm reads in DICOM medical imaging files and applies various image processing techniques to segment and extract objects of interest from the image. The image processing pipeline involves several steps, including adaptive thresholding, morphological filtering, gradient magnitude calculation, watershed transform, marker identification, and segmentation. Figures 6–9 illustrate the different steps involved in the image processing pipeline. The input pre-processed images using adaptive thresholding and morphological filtering to remove small objects from the image. The region properties of the remaining objects are then calculated, and the gradient magnitude image is computed. The watershed transform is applied to segment the image based on its gradient magnitude, and morphological operations are performed to improve the segmentation results. The regional maxima are identified, and the image is thresholded to extract markers for the watershed segmentation. The distance transform is computed to assign values to each pixel based on its distance to the nearest boundary. Finally, the watershed segmentation is performed, and the regions in the image are labelled, and the labelled image is superimposed onto the original image to display the regions of interest. The figures provided in the context demonstrate the different stages of image segmentation using various processing techniques, including;

- Gradient magnitude
- Watershed transform of gradient magnitude
- Regional maxima superimposed on original image
- Modified regional maxima superimposed on original image
- Markers and object boundaries superimposed on original image
- Coloured labels superimposed transparently on original image

**Figure 10:**
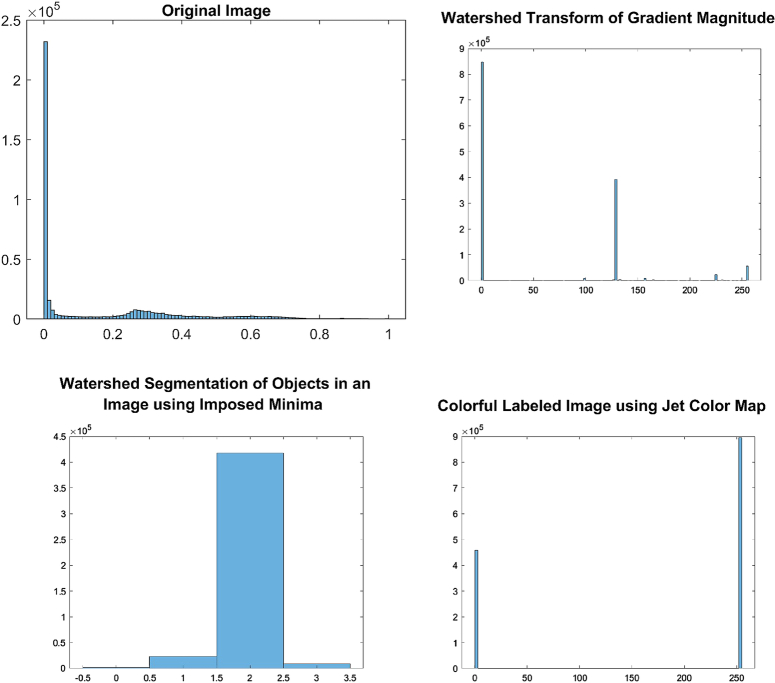
Displays histograms for various stages of image processing for patient 1. The histograms show the distribution of pixel intensities for the original pre-processed image, the watershed transform of the gradient magnitude image, the watershed segmentation of objects using imposed minima, and the labelled image using a jet colour map.

**Figure 11:**
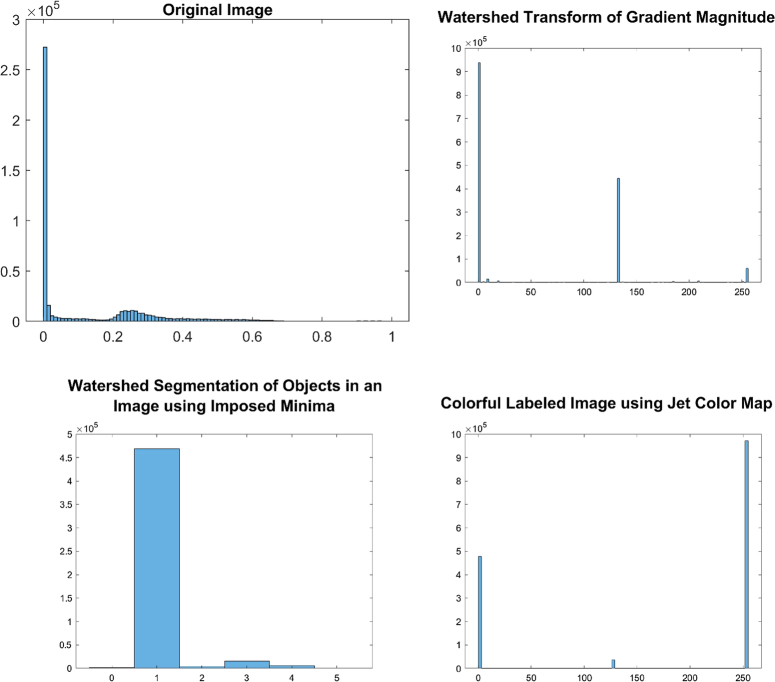
Displays histograms for various stages of image processing for patient 2. The histograms show the distribution of pixel intensities for the original pre-processed image, the watershed transform of the gradient magnitude image, the watershed segmentation of objects using imposed minima, and the labelled image using a jet colour map.

**Figure 12:**
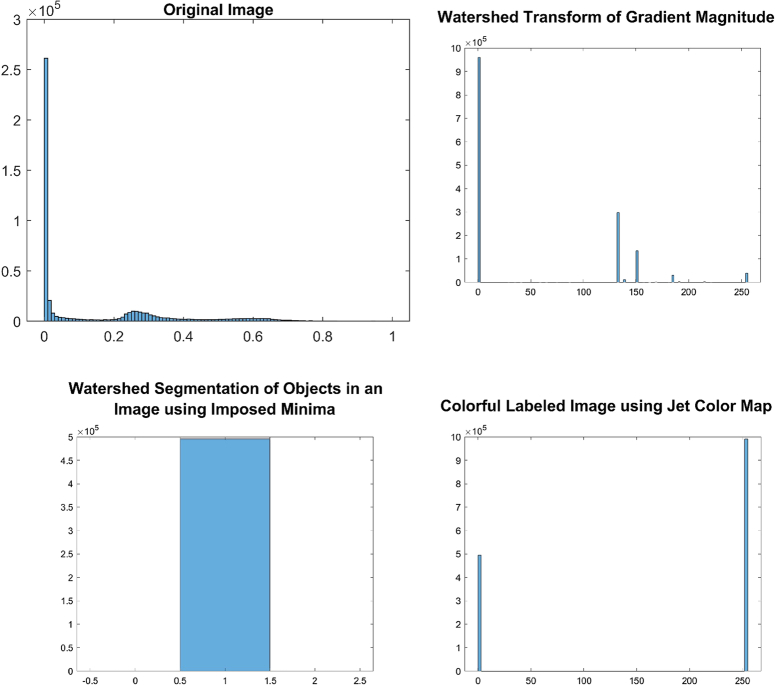
Displays histograms for various stages of image processing for patient 3. The histograms show the distribution of pixel intensities for the original pre-processed image, the watershed transform of the gradient magnitude image, the watershed segmentation of objects using imposed minima, and the labelled image using a jet colour map.

**Figure 13:**
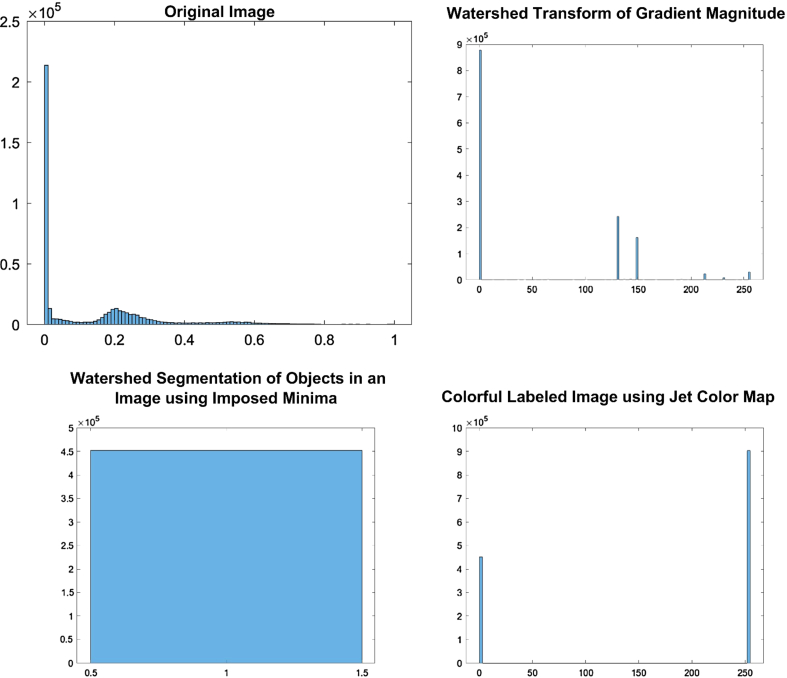
Displays histograms for various stages of image processing for patient 4. The histograms show the distribution of pixel intensities for the original pre-processed image, the watershed transform of the gradient magnitude image, the watershed segmentation of objects using imposed minima, and the labelled image using a jet colour map.

The implementation of the given algorithm for DICOM medical imaging data involves numerous steps of image processing techniques to extract objects of interest from the image. The figures provided in the context outline the various steps in the image segmentation pipeline, including adaptive thresholding, morphological filtering, gradient magnitude calculation, watershed transform, marker identification, and segmentation. In order to provide further analysis of the pixel intensities within the image, we have also included histograms of input pre-processed images and the final labelled image. These histograms plot the frequency of pixel intensities within the image, which helps to analyze the distribution and variability of these intensities. The titles of the histograms are “original image,” “watershed transform of gradient magnitude,” “watershed segmentation of objects in an image using imposed minima,” and “colourful labelled image using jet colour map” (Figs. 10–13). This information is useful in identifying specific image features and objects, as well as in comparing the results with other images. By examining the histograms alongside the image processing figures, the reader can better understand the relationship between the intensity of individual pixels and their significance in determining the presence of objects of interest within the image. The combination of the images and histograms provides an in-depth analysis of the image processing pipeline and demonstrates the potential for improved medical imaging diagnostics using advanced image processing techniques.

The histograms generated for the processed images reveal insightful information about their characteristic features. The histogram of the original image displays a roughly normal distribution of pixel values, which is useful in estimating the mean and variance of the pixel values. The histogram of the watershed transform of the gradient magnitude reveals the distribution of edges in the image, which can be used to identify the boundaries of objects. The histogram of the watershed segmentation of objects using imposed minima shows the distribution of object sizes, and it is useful in filtering out objects of a specific size. Finally, the histogram of the labelled image shows the distribution of colours assigned to each object in the image, which makes it easier to distinguish between different objects. These histograms provide useful information to optimize the image processing pipeline and make informed decisions in image analysis.

**Figure 14:**
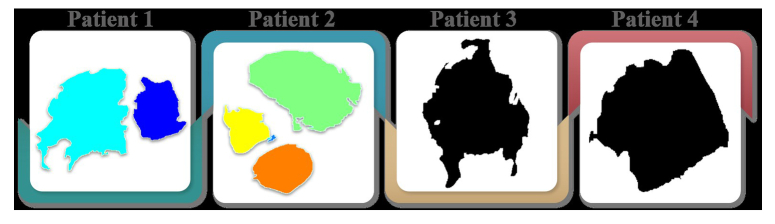
Glioblastoma multiforme tumours in four patients were segmented.

The MCWS technique was effective in correctly extracting GBM tumours from all four patients, regardless of the number and location of the tumours (Fig. 14). For patients 1 and 2, the tumours were segmented by overlaying transparently coloured labels on the original or “f” images with the gradient magnitude or “a” images. However, for patients 3 and 4, the line that is typically present in the watershed algorithm was absent in images “e”, which required the use of different methods, such as displaying the gradient magnitude, to extract the tumour. Nevertheless, the tumours were successfully extracted in all four patients using various methods.

The results indicate that the mean sensitivity for GBM segmentation was 0.9905 (99.05%), indicating that the model was successful in correctly identifying GBMs in most cases. Similarly, the Specificity was 0.9483, which means that the model was effective in identifying cases without GBMs in most cases. The Accuracy, which is a combination of Sensitivity and Specificity, was 0.9508, indicating that the overall performance of the model in GBM segmentation was good. Although the precision value is 0.5481, the total number of true positive samples identified by the algorithm is efficient in overall GBM detection (Table 3).

**Table 3:** Characteristics performance metrics for GBM segmentation.

	Sensitivity (or recall)	Specificity	Accuracy	Precision	F_Measure	Jaccard
Figure 2, Patient 1	0.9890	0.9461	0.9492	0.5905	0.7395	0.9049
Figure 3, Patient 2	0.9903	0.9538	0.9557	0.5399	0.6988	0.8998
Figure 4, Patient 3	0.9835	0.9432	0.9459	0.5472	0.7032	0.9602
Figure 5, Patient 4	0.9992	0.9501	0.9525	0.5149	0.6796	0.9714
Mean	0.9905	0.9483	0.9508	0.5481	0.7052	0.9340

GBM, glioblastoma multiforme.

The F-Measure is an essential tool for evaluating the efficacy of a classification model in classifying data accurately. By producing a score ranging between 0 and 1, it provides an indication of overall accuracy, with higher scores indicating better model performance. In the study, the classification model under analysis exhibits reasonable accuracy, with F-Measure scores ranging from 0.6796 to 0.7935. The highest F-Measure score reflects the model’s strong precision and recall, while the lowest score indicates slightly reduced precision and recall. Such findings indicate that the classification model can effectively identify and categorize data, albeit with some variability.

The Jaccard similarity coefficient is a useful metric for evaluating the efficacy of a classification model, particularly when working with imbalanced datasets. The coefficient measures the similarity between the predicted and actual result sets, producing a score between 0 and 1. Higher values reflect greater similarity, with a perfect score of 1.0 indicating an accurate the classification model. In interpreting the Jaccard values provided, it is clear that the classification model has produced relatively high similarity scores, ranging from 0.8998 to 0.9714, indicating that the model is effectively identifying true positive and negative cases. These Jaccard values suggest that the model is reasonably accurate and may be applied successfully in various contexts.

In total, the proposed algorithm is efficient and reliable in GBM segmentation, proving to be a promising approach for automated tumour detection from MRI images. Our approach’s effectiveness is attributed to the MCW algorithm employed, enhancing the segmentation quality of the MRI images and identifying the glioblastoma multiforme regions with higher accuracy. Using this efficient MCW algorithm, we can diagnose tumour patients quickly and accurately, providing timely treatment and resulting in improved patient outcomes.

In medical image analysis, both speed and accuracy are crucial for the effective diagnosis and treatment of GBM tumours. The importance of prioritizing speed based on response time, as evidenced by the user of the system, cannot be understated. However, it is crucial to note that accuracy should not be sacrificed in the quest for speed. Striking a balance between the two factors is necessary to achieve optimal results. In this context, the MCWS algorithm provides an effective approach for automating the segmentation of GBM from MRI images while achieving higher segmentation accuracy. The response time demonstrated in the study, which is equal to 6.2335 seconds (RAM: 16.0 GB and CPU: Intel(R) Core(TM) i5-7500 CPU @ 3.40GHz), underscores the importance of a time-efficient approach to medical image analysis.

## Discussion

The successful segmentation of GBM tumours in all four patients using the MCWS technique demonstrated the effectiveness of this approach in the segmentation of medical images. An important aspect of this approach is that it lacks dependence on educational data, meaning that it does not require manually labelled data to train the algorithm. The technique was able to accurately extract tumours regardless of their number, shape, and location in the brain. This is a significant advancement in medical imaging, as GBM tumours are notoriously difficult to treat due to their invasive and heterogeneous nature. The MCWS technique provides a reliable method for accurately identifying the boundaries of the tumour, which can improve treatment planning and patient outcomes.

The high soft tissue resolution in MRI images has led to their suggestion as a valuable tool for segmentation in multiple previous studies^32–35^. While semi-automatic^35,36^ and automated^26,27,33^ segmentation methods for brain tumour segmentation, particularly for GBM, have proven useful, fully automatic techniques such as the MCWS method hold greater promise due to their independence from the image’s spatial resolution^17,18,37,38^. Our technique, which utilizes fully automatic methods, was able to completely extract GBM tumours in all cases (100%), demonstrating the efficacy of fully automatic methods in achieving segmentation without dependence on image resolution. Furthermore, our approach displayed high and consistent levels of sensitivity, accuracy, and specificity when compared to previous studies^39–41^.

In this study, we have demonstrated the simultaneous and sequential use of FCM and MCWS techniques for the first time in GBM extraction, leading to complete tumour segmentation. An accurate diagnosis of tumour size, regardless of the location of the tumour in the brain, was achieved with high accuracy and sensitivity by finding the edges of the tumour from the oedema section. The ability to accurately distinguish the edges of the tumour, especially from the brain cortex, was a significant advantage of our method. Furthermore, several different areas of the tumour could be accurately separated.

The parameters of characteristic performance metrics, including sensitivity, specificity, validity, and accuracy, were calculated and compared with previous works, and the results show that the use of the MCWS technique with FCM thresholding without using pre-processing methods such as motion correction and denoising is the best possible ratio. This method also showed superiority and speed, as evidenced by the response time of 6.2335 s. In comparison, the response time for the watershed method was reported as 9.219 s in previous work^42^. However, as seen in the segmented images 10 and 11 (patients no. 2 and 3), some normal brain anatomy was not recognized due to the pressure of the tumour on the midline of the brain and the shift of the brain to the side. Nevertheless, the use of the MCWS technique was a prominent point in our method.

Our study also demonstrated the importance of an accurate diagnosis for a better prognosis and diagnosis of GBM. The use of the MCWS technique with T2-w images instead of images with T1-weighted post-contrast injection was suggested due to the lack of invasiveness and additional cost of injection in T2-w images and the high accuracy of tumour detection compared to the conventional injection method and T1-w post-imaging. Our method can potentially improve the diagnostic process for GBM and reduce the cost and invasiveness associated with traditional diagnostic methods.

Our study has important implications for specialists in radiology, radio-oncology, neurology, and neurosurgery in the accurate diagnosis of GBM for better prognosis and diagnosis. However, to further validate our findings, we suggest conducting prospective longitudinal and cross-sectional studies involving a larger range of patients with tumours and different grades of the disease. It is also recommended to examine different brain tumours, especially GBM, with the same method in 3D images as well as in other coronal and sagittal sections to calculate the volume, gender, and consistency of the tumour. Overall, our study demonstrates a novel and effective method for the accurate diagnosis and segmentation of GBM tumours, with potential applications in improving patient outcomes and informing clinical decision-making.

Artifacts and noise are commonly observed in medical images, including MRI scans^43^. These can be caused by various factors such as patient motion during imaging, hardware limitations, and external interference^43,44^. The presence of artifacts can always be destructive and interfere with the accurate interpretation of images. However, the MCWS technique used in the study has been shown to be effective even in the presence of movement artifacts. This is because the technique uses a gradient-based approach that can differentiate between the edges of the tumour and the artifacts. It should be noted that applying the denoising process to medical images is not always desirable. Although it can help to remove noise, it can also remove important information related to changes in spatial resolution. This can interfere with the process of segmentation and finding the tumour’s edges. Therefore, it is important to carefully consider the balance between removing noise and preserving important image features when applying denoising techniques to medical images. In sum, the proper management of noise and artifacts is critical for accurate image interpretation and the successful diagnosis and treatment of medical conditions (see supplementary file 1, Supplemental Digital Content 2, http://links.lww.com/MS9/A365).

In addition, the field strength of MRI devices can also affect the quality of the images obtained. Higher field strengths can provide better image resolution, but they can also increase image noise and artifacts. Therefore, it is important to carefully consider the appropriate field strength when performing medical imaging studies.

The study conducted for this research was limited by the small sample size of only four patients. While our study demonstrated promising results, it is important to conduct prospective longitudinal and cross-sectional studies to investigate a larger range of patients with tumours and different grades of the disease. This will help validate the effectiveness and reliability of the method in a larger population and provide more insights into the method’s limitations and potential for further improvement. It is also suggested to examine different brain tumours, especially GBM, with the same method in three-dimensional images as well as in other coronal and sagittal sections to calculate the volume, sex, and consistency of the tumour. These additional studies can provide more comprehensive information about the tumour and help guide treatment decisions.

## Conclusion

In conclusion, we proposed a novel method for GBM segmentation from T2-w MRI images using the MCWS technique. This method demonstrated high accuracy and efficiency, with significantly reduced response time compared to the watershed method. Our study contributes to the current body of research on GBM segmentation and has potential clinical implications for improving the accuracy and sensitivity of GBM diagnosis and treatment planning. Our research highlights the potential clinical implications of automated GBM segmentation and contributes to the development of advanced image processing methods for radiology and neuro-oncology. Ultimately, future studies could build on our findings to further refine and optimize the MCWS algorithm for other brain tumour segmentations.

## Ethics statement and consent for publication

Our institutional ethical committee does not require ethical board approval from case series as long as researchers can provide an informed consent form. Written informed consent was obtained from the patient for publication of this case report and accompanying images. A copy of the written consent is available for review by the Editor-in-Chief of this journal on request.

## Consent

Written informed consent for this case series was obtained for publication. A copy of the written consent is available for review by the Editor-in-Chief of this journal.

## Source of funding

This research work was conducted without any external funding.

## Author contribution

Conceptualization: S.Gh., S.M., K.Gh.; Methodology: S.Gh., K.Gh.; Software: S.Gh., S.M., K.Gh., F.K.; Validation: S.Gh., S.M., K.Gh.; Formal analysis: S.Gh.; Investigation: S.Gh., S.M.; Resources: S.Gh., M.M.; Data curation: S.Gh., S.M., M.M.; Writing—original draft: S.Gh., S.M.; Writing—review and editing: S.Gh., S.M., K.Gh.; Visualization: S.Gh.; Supervision: S.Gh.; Project administration: S.Gh., S.M., K.Gh.

## Conflicts of interest disclosure

No conflict of interest was identified by the authors.

## Research registration unique identifying number (UIN)

Not applicable.

## Guarantor

Dr Kayvan Ghaderi.

## Provenance and peer review

Not commissioned, externally peer-reviewed.

## Data availability statement

This article contains all of the data produced or analyzed during this investigation. Any further inquiries should be forwarded to the corresponding author.

## Supplementary Material

SUPPLEMENTARY MATERIAL
