# Supplementary material for: Marker-controlled watershed algorithm and fuzzy C-means clustering machine learning: automated segmentation of glioblastoma from MRI images in a case series
Source: Ann Med Surg (Lond). 2024 Jan 26;86(3):1460–75. doi: 10.1097/MS9.0000000000001756 (PMC10923355; doi:10.1097/MS9.0000000000001756)
Supplement: SUPPLEMENTARY MATERIAL [file ms9-86-1460-s002.docx]

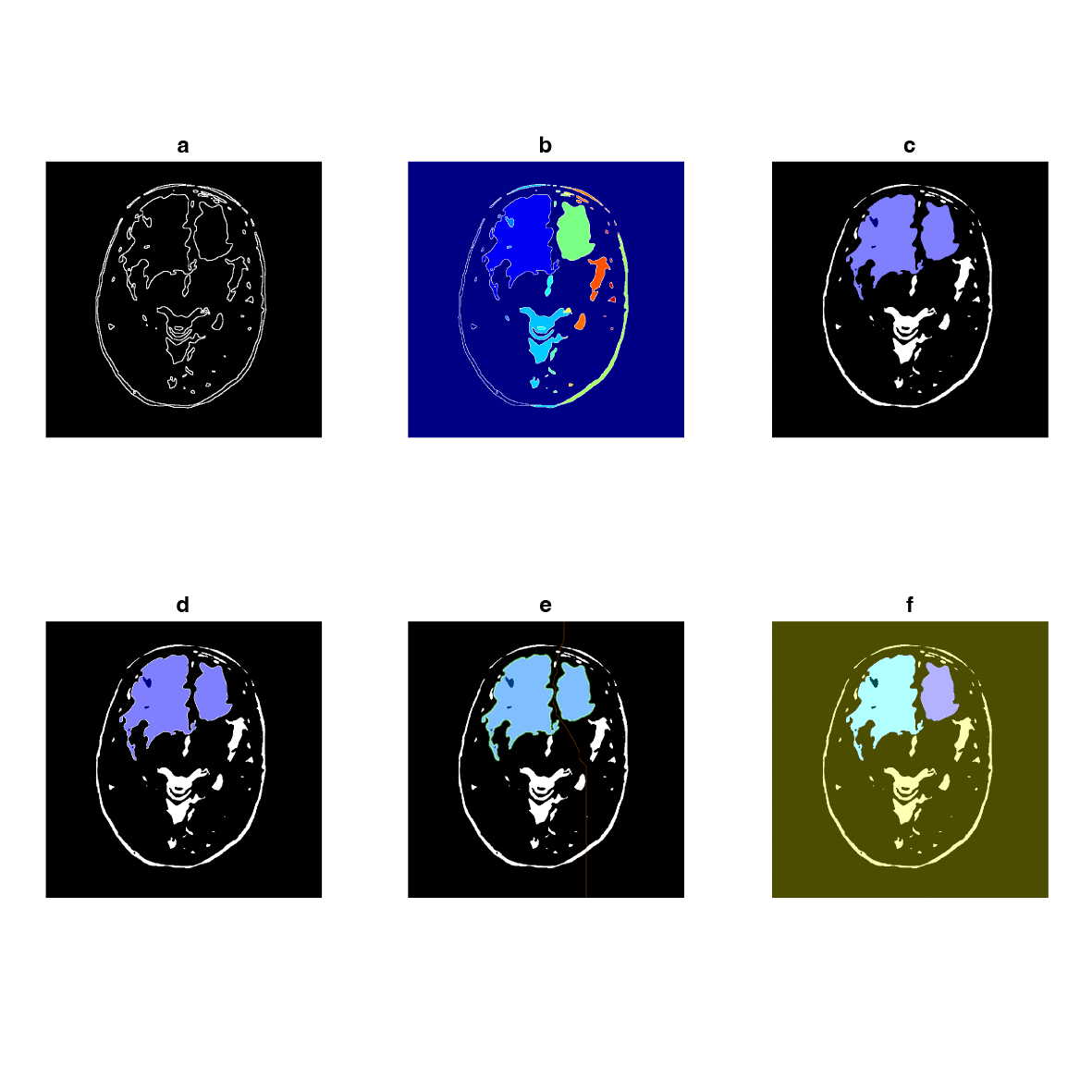


**Figure 1.** The MCWS algorithm for GBM segmentation with Gaussian filter for image denoising (patient 1)


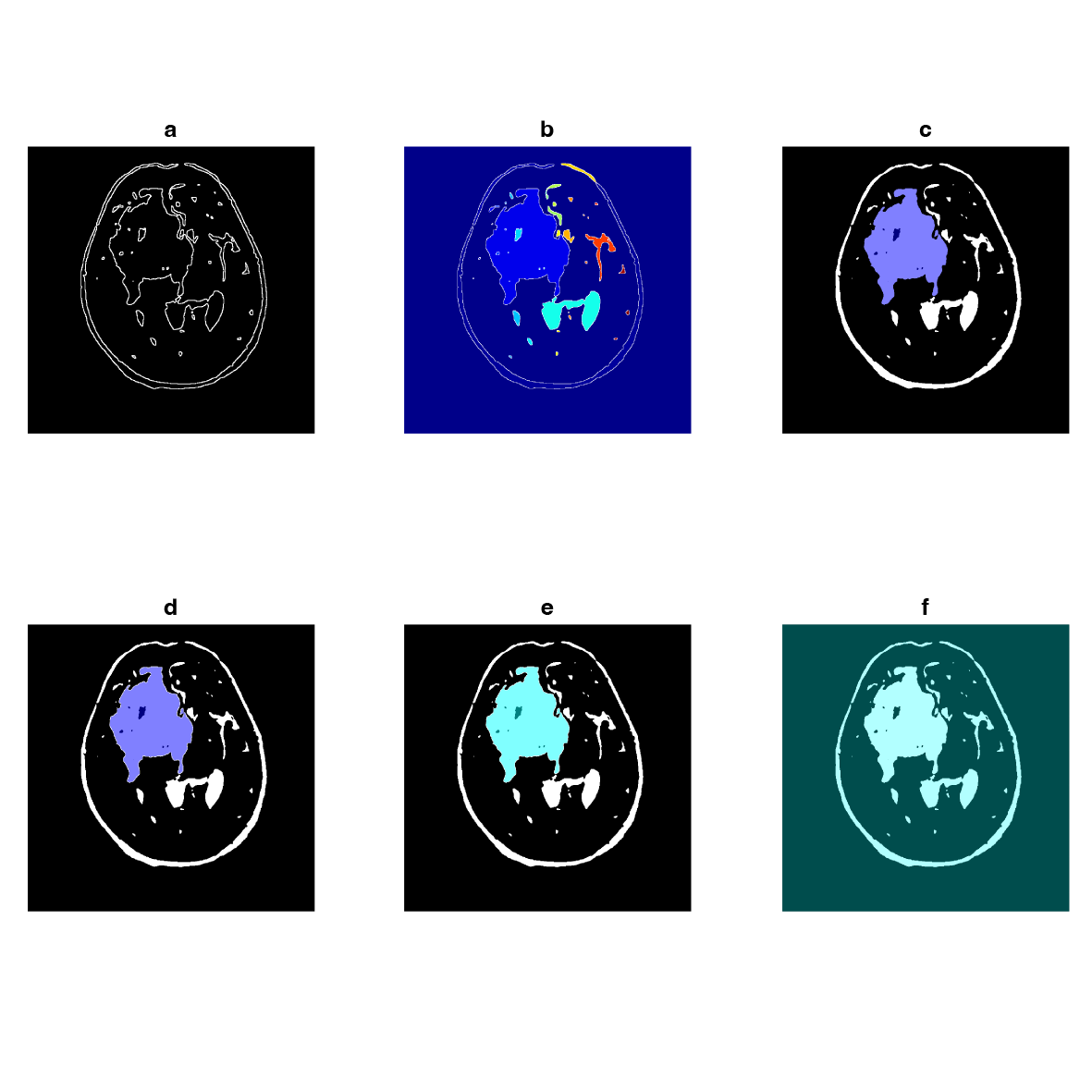


**Figure 2.** The MCWS algorithm for GBM segmentation with Gaussian filter for image denoising (patient 2)


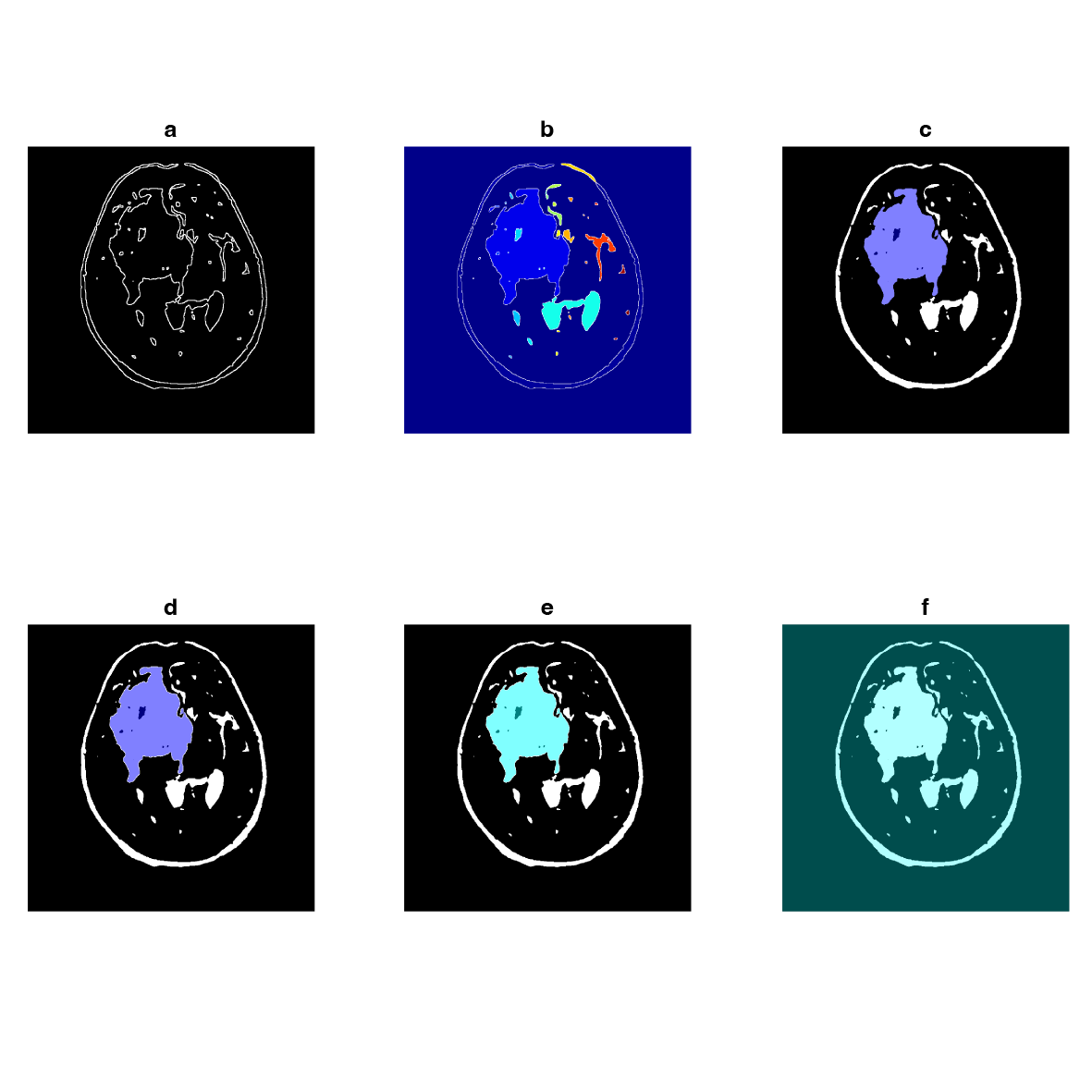


**Figure 3.** The MCWS algorithm for GBM segmentation with Gaussian filter for image denoising (patient 3)


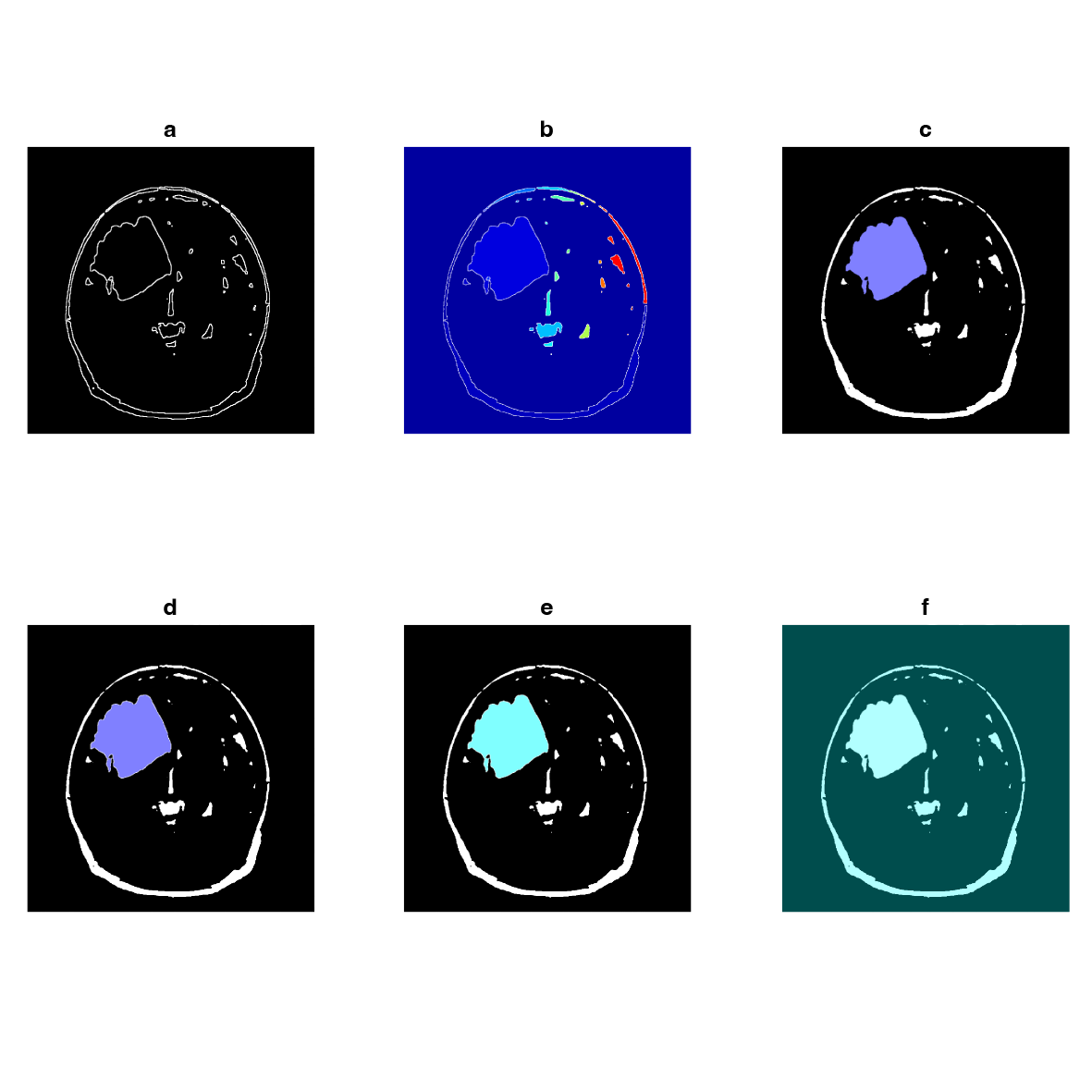


**Figure 4.** The MCWS algorithm for GBM segmentation with Gaussian filter for image denoising (patient 4)

Apply Gaussian filter for image denoising:

| **Table 1.** Thresholding levels | | | |
| --- | --- | --- | --- |
| # | FCM Threshold Level 0 | FCM Threshold Level 1 | Otsu Level |
| Figure 5, Patient 1 | 0.1576 | 0.4627 | 0.2196 |
| Figure 6, Patient 2 | 0.1351 | 0.3795 | 0.1686 |
| Figure 7, Patient 3 | 0.1472 | 0.4348 | 0.2000 |
| Figure 8, Patient 4 | 0.1193 | 0.3847 | 0.1569 |
